# Postpartum modern contraceptive use in northern Ethiopia: prevalence and associated factors - methodological issue in this cross-sectional study

**DOI:** 10.4178/epih.e2017019r

**Published:** 2017-05-10

**Authors:** Teklehaymanot Huluf Abraha, Alemayehu Shimeka Teferra, Abebaw Addis Gelagay

**Affiliations:** 1 Department of Public Health, College of Health Sciences, Aksum University, Aksum, Ethiopia; 2 Department of Epidemiology and Biostatistics, College of Medicine and Health Sciences, University of Gondar, Gondar, Ethiopia; 3 Department of Reproductive Health, College of Medicine and Health Sciences, University of Gondar, Gondar, Ethiopia

We greatly respect the comments made by the above readers of our article, as they have precisely pointed out a methodological limitation of our design. We certainly recognize this study design limitation. In our nation (Ethiopia), the prevalence and associated factors of postpartum contraceptive use have not yet been assessed. Therefore, we as the authors of this study believe that assessing the magnitude of modern contraceptive use and its determinants using a cross-sectional study design is very important in the study area.

Moreover, cross-sectional studies provide an ample data that are useful for planning health services and medical programs. Our study measured the prevalence of postpartum modern contraceptive use (48.0%), and examined the relationship between the outcome variable (postpartum modern contraceptive use) and associated factors as they existed in the defined population at a particular time. However, a longitudinal study would not address the prevalence. The interpretation of the results of this article does not change based on this aspect of our research. Despite this limitation of the study design (i.e., the fact that it lacks a temporal relationship), we believe that the advantages of a cross-sectional study design outweigh its limitations. We also believe that this limitation can be overcome in the near future by changing the study design. Moreover, we as the authors have addressed this issue in our research objectives.

The main objective of our article was to assess the prevalence and associated factors of modern contraceptive use in the town of Aksum, northern Ethiopia. We, the authors, are confident that the many scholars who are readers of this journal will find value in our article.

